# Economic and biological costs of cardiac imaging

**DOI:** 10.1186/1476-7120-3-13

**Published:** 2005-05-25

**Authors:** Eugenio Picano

**Affiliations:** 1 CNR, Institute of Clinical Physiology, Pisa, Italy

## Abstract

Medical imaging market consists of several billion tests per year worldwide. Out of these, at least one third are cardiovascular procedures. Keeping in mind that each test represents a cost, often a risk, and a diagnostic hypothesis, we can agree that every unnecessary and unjustifiable test is one test too many. Small individual costs, risks, and wastes multiplied by billions of examinations per year represent an important population, society and environmental burden. Unfortunately, the appropriateness of cardiac imaging is extra-ordinarily low and there is little awareness in patients and physicians of differential costs, radiological doses, and long term risks of different imaging modalities. For a resting cardiac imaging test, being the average cost (not charges) of an echocardiogram equal to 1 (as a cost comparator), the cost of a CT is 3.1x, of a SPECT 3.27x, of a Cardiovascular Magnetic Resonance imaging 5.51x, of a PET 14.03x, and of a right and left heart catheterization 19.96x. For stress cardiac imaging, compared with the treadmill exercise test equal to 1 (as a cost comparator), the cost of stress echocardiography is 2.1x and of a stress SPECT scintigraphy is 5.7x. Biohazards and downstream long-term costs linked to radiation-induced oncogenesis should also be considered. The radiation exposure is absent in echo and magnetic resonance, and corresponds to 500 chest x rays for a sestamibi cardiac stress scan and to 1150 chest x rays for a thallium scan. The corresponding extra-risk in a lifetime of fatal cancer is 1 in 2000 exposed patients for a sestamibi stress and 1 in 1000 for a thallium scan. Increased awareness of economic, biologic, and environmental costs of cardiac imaging will hopefully lead to greater appropriateness, wisdom and prudence from both the prescriber and the practitioner. In this way, the sustainability of cardiac imaging will eventually improve.

## The unbearable lightness of being [a cardiac imaging specialist]

A Renaissance of cardiac imaging occurred in the 1980s [1]. New technologies allowed the non-invasive description of cardiac function, perfusion, and metabolism in a polychrome, three-dimensional, overwhelming fashion. Almost unlimited resources were devoted to patient care in the economic framework of the affluent society. At the beginning of the 1990s, The Renaissance made its transition into the splendid decadence of the Baroque. The increasing technological burden in clinical cardiology paradoxically did not bring a parallel increase in the quality of care but rather an increase in cost. The economic climate had changed; the illusion of unlimited economic resources had come to an end [2]. Keeping in mind that each test represents a cost, often a risk, and always a diagnostic hypothesis, we can agree that every unnecessary and unjustifiable test is one test too many. Small individual costs, risks, and wastes multiplied by billions of examinations per year represent an important population [3], society [4] and environmental [5] burden. Unfortunately, the appropriateness of cardiac imaging is usually extra-ordinarily low and there is little awareness among patients and physicians of the elementary physical basis, differential costs, radiological doses, and long term risks of different imaging modalities [6]. It is also well known that – in the words of Bernard Lown – "*technology in medicine is frequently untested scientifically, often applied without data relating to cost benefit, and driven by market forces rather than by patient needs*." Bernard Lown, 2004 [7].

## The cost of cardiac imaging

**Figure 1:**
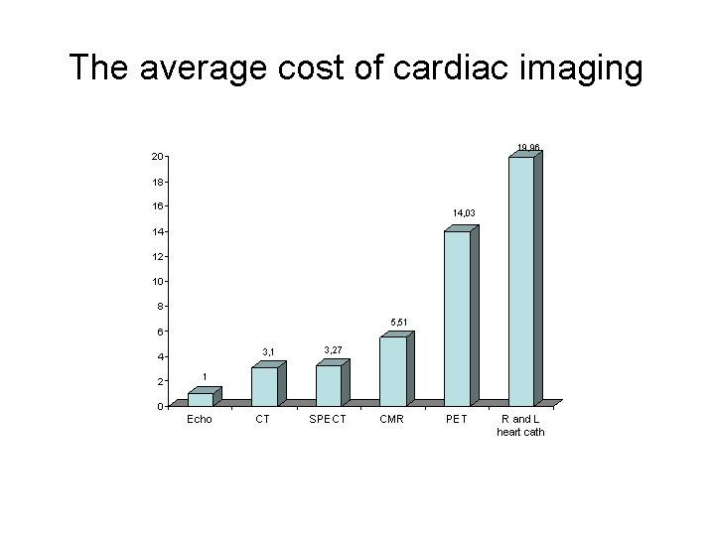
The average costs of CMR and other common cardiac imaging procedures when compared 2 D echocardiography (modified from Pennell, ref. 12).

*"Ten years ago, medical imaging wasn't even in the radar screen for most health insurers. In 2004, it' s one of the highest cost items in a health plan's medical budget, and also one of the fastest growing"*. (Atlantic info service newsletter, 2004) [8]. As an example, in U.S. during the year 2002, 7.8 million cardiac perfusion scans were performed, with a growth of 40% in the last 3 years [9]. Still in U.S., about 10 million CT scans were done in 1993, and about 60 millions in 2001 [10]. Booz Allen Hamilton projects that spending on diagnostic imaging could grow 28 % by 2005, with utilization growing by 9% per year. No doubt that any assessment of costs of medical imaging should also include the often unquestionable downstream benefits, leading to a reduction of overall costs due to more disabling disease prevented. For instance, ultrasound screening for abdominal aneurysms can reduce the risk of death by more than 50 % in men aged 65–74. When projected to 10 years, that screening costs 13.000 dollars per life year gained – a highly favourable result in terms of cost-effectiveness [11]. However, it is beyond question that the explosion in imaging costs is also driven by a number of factors which not always improve the cost-effectiveness. High patient demand for the newest diagnostic tests, physicians eager to use the most effective technologies, and possibly even some providers who may boost utilization to help pay off investments in high-tech equipment. All too often physicians may not have adequate controls on the number of high cost imaging tests they are ordering. For a resting cardiac imaging test, being the average cost (not charges) of an echocardiogram equal to 1 (as a cost comparator), the cost of a CT is 3.1x, of a SPECT 3.27x, of a Cardiovascular Magnetic Resonance imaging 5.51x, of a PET 14.03x, and of a right and left heart catheterization 19.96x (Fig 1) [12]. For stress cardiac imaging, compared with the treadmill exercise test equal to 1 [as a cost comparator], the cost of stress echocardiography is 2.1x and of a stress SPECT scintigraphy is 5.7x [13]. Obviously, the evaluation of costs of medical imaging should also imply the entire clinical context (pre-test likelihood of disease, basic risk profile, target of stress testing, socio-economic characteristics of the health care system, etc.) to be taken into account. Some measures of precision and/or dispersion of each modality as compared to echocardiography may provide a more comprehensive information in the specific health care milieu where these tests are actually performed.

## The individual and social risks of imaging

**Figure 2:**
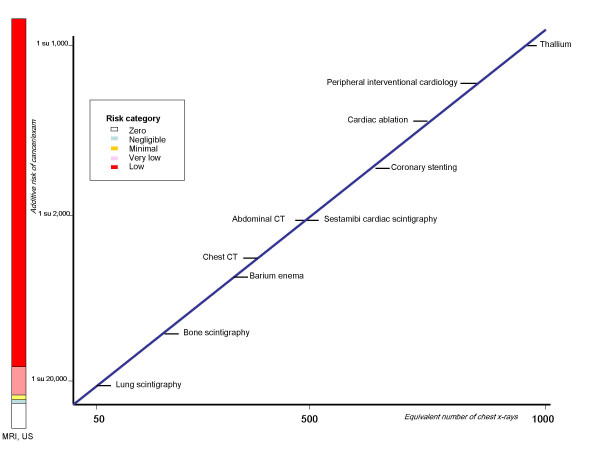
Presentation of cancer risk and radiation dose (in multiples of dose from a simple chest x rays) for some common radiological and nuclear medicine examinations (Modified from Picano E, ref. 37)

Current protection standard and practices are based on the premise that any ionising radiation dose, no matter how small, can result in detrimental health effects [14]. These include long-term development of cancer and genetic damage [15]. For the purposes of radiation protection, the dose-response curve for radiation-induced cancer is assumed to be linear at low doses, with no minimum threshold [16]: (Fig. 2). The dose of 50 chest X rays (for example, a lung scintigraphy) corresponds to an extra-risk of cancer of about 1 in 20,000 exposed patients. The dose of 500 chest x rays (such as technetium sestamibi scan) corresponds to an extra-risk of about 1 in 2000 exposed patients. The dose of 1 000 chest x rays (associated with a Thallium scan) corresponds to an extra risk of cancer of about 1 in 1 000 exposed patients [17]. The radiation dose and risk associated with some common imaging examinations are expressed in Table 1 as equivalent dose of natural yearly background radiation, extra-risk of fatal cancer in the lifetime and lost life expectancy per exam [18]. Presented data refer to the best available estimates from the radiological Commission of Radiation Protection and conform to their suggested standards for communicating risk to patients. These estimations are a benchmark for the physicians and are incorporated in the European Commission guidelines for medical imaging. These small individual risks multiplied by billion examinations become significant population risk.

**Table 1:** Radiation doses and estimated cancer risk from common radiological examinations and isotope scans

**Type of test**	**Effective radiation dose (mSv)**	**Equivalent period of natural background radiation**	**Lifetime additional risk of cancer/examination**	**Lost life expectancy**	**Equivalent n. of chest x-rays**
Chest radiograph	0.01	A few days	Negligible risk	2 minutes	1
Skull radiograph	0.1	A few weeks	Minimal risk (1 in 100,000 to 1 in1,000,000)	20 minutes	5
Lung isotope scan	1	A few months to a year	Very low risk (1 in 10,000 to 1 in 100,000)	3 hrs	50
Cardiac gated study	10	A few years (4 years)	Low risk (1 in 2,000)	2 days	500
Thallium scan	20	(8 years)	(1 in 1,000)	4 days	1000

Use of radiation for medical examinations and test is the largest manmade source of radiation exposure. The medical sources of radiation were about one fifth of the natural radiation in 1987 [18], close to one-half in 1993 [19], and almost 100% of natural radiation in 1997 [20], and the use of procedures with a high load of radiation continues to grow steadily [21].

This impressive amount of ionizing friendly fire translates into a significant population risk. Lifetime risk of developing cancer attributable to diagnostic X-rays is 0.6–3.2% in developed countries [22]. The numbers are striking, but underestimate the biological population burden of medical radiation for three reasons. First, they refer to the radiological volume of 10 years ago – substantially lower than the current radiological volume. Second, they do not consider the practice of nuclear medicine, which adds a further 10 % to the global radiation burden. Third, in addition to the risk of cancer, one should consider the burden of teratogenesis. The risk of radiation – induced damage passed onto the offspring is estimated to represent a fifth of the risk of fatal cancer [23].

## Unawareness in imaging

**Figure 3:**
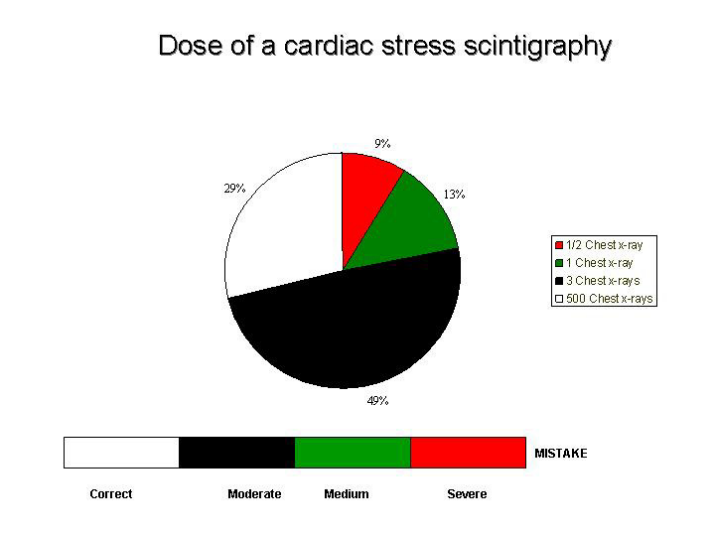
What cardiologists know about dose of a test they prescribe and/ or perform daily. 2 out of 3 physicians underestimate of 100 to 500 times the dose of a cardiac scintigraphy. The dose is equivalent to 500 chest x rays with technetium sestamibi, and to more than 1000 chest x rays with thallium scan (modified from Correia et al, ref 28)

Several recent studies clearly prove that not only general practitioners but also cardiologists, orthopaedics and even radiologists and nuclear physicians usually ignore the dose and the risk of what they do: and the more they do, the more they tend to ignore [24-28]. Radiologists working in an academic US environment frequently underestimate of 100 to 500 times the dose of a common CT and 97 % of UK doctors underestimate of sixteen times the dose of a common CT chest scan. In 1 case out of 10, doctors believe that magnetic resonance employs ionising radiations, and 1 case out of 20 that ultrasound employs ionising radiation [25,27]. The majority of doctors, and even of radiologists, is not aware of the oncogenic risk of common, high dose radiological examinations [26,28]. The vast majority of cardiologists underestimates of 200 up to 1000 times the dose of a stress cardiac perfusion scintigraphy [28]: Figure 3. This unawareness generates inappropriateness, which is an endemic and pervasive disease in the world of cardiac imaging. We need more prudence, wisdom and responsibility in indicating and performing (cardiac) imaging tests. Prudence and responsibility should obviously be especially high for more expensive tests, greater for those exposing the patient to significant ionizing radiation [29], and greatest in special subset particularly vulnerable to the damaging effects of ionizing radiation, such as women in reproductive age [30] and children [31-33].

## From benefit to risk-benefit

According to the International Commission of Radiological Protection "Medical exposure is the only category in which large reductions in average dose are possible, and it is therefore highly desirable to reduce applications of medical radiation which are of no benefit to the patients and to minimise useless radiation in the course of medical examinations" [34]

In other words it is desirable to adopt a radiation sparing strategy not only for the physician and the patients but also for the society and the environment. Nuclear medicine and X-ray procedures are intended, as stated by the United Nations Annex on Medical Exposures, "to provide doctors with diagnostic information and in principle conducted with the lowest practicable levels of patient dose to meet clinical objectives" [5]. The clinical counterpart of this concept is that it is not enough that a test is marginally "better" than the other to justify its use: the extra-value should be proportional to the extra-cost and to the extra-risk. Both the physician and the patient should be well aware of the different individual risks and social costs posed by different diagnostic options. As recently stated by guidelines on fluoroscopically guided invasive cardiovascular procedures, " the core principle governing the use of ionizing radiation is ALARA (as low as reasonably achievable). The ALARA principle recognizes that there is no magnitude of radiation exposure that is known to be completely safe. This principle confers a responsibility on all physicians to minimize the radiation injury hazard to their patients, to their professional staff, and to themselves" [35]. If the information is comparable, every effort should be done to orient the patient towards non-ionizing testing. If it is true that "local expertise and availability should guide the selection of imaging techniques" [36], it is also true that the doses and risks associated with the different diagnostic options should be clearly spelled out to allow the patient and the prescriber to make an informed decision. This policy is encouraged by common sense [37], deontologic code [38], European Commission imaging guidelines [14] and the European law [39]. It propels to use a green technique whenever the information supplied is grossly comparable to the red one [40].

The basic concept underlying this cardiac imaging paradigm shift is obvious but is presently neglected. Medical images of heartbreaking beauty when considering the benefit, can be ambiguous when considering the cost-benefit relationship, and unacceptable when considering the risk-benefit. In this transition, we as physicians and imaging specialists, are the critical link. *"Health professionals involved in the processes of diagnosis and treatment are the critical link. Training them properly and ensuring intensive information exchange among them are, therefore, probably the most cost-effective ways of achieving patient safety*" [4]. (International Action Plan for the radiological Protection of patients).
